# Decisional needs of patients with recurrent high-grade glioma and their families

**DOI:** 10.1093/nop/npac046

**Published:** 2022-06-11

**Authors:** Helle Sorensen von Essen, Dawn Stacey, Karina Dahl Steffensen, Rikke Guldager, Frantz Rom Poulsen, Karin Piil

**Affiliations:** Department of Neurosurgery, Odense University Hospital, Odense, Denmark; Clinical Institute and BRIDGE (Brain Research-Interdisciplinary Guided Excellence), University of Southern Denmark, Odense, Denmark; Center for Shared Decision Making, Region of Southern Denmark, Vejle, Denmark; Department of Regional Health Research, Faculty of Health Sciences, University of Southern Denmark, Odense, Denmark; School of Nursing and Ottawa Hospital Research Institute, University of Ottawa, Ottawa, Ontario, Canada; Center for Shared Decision Making, Region of Southern Denmark, Vejle, Denmark; Department of Regional Health Research, Faculty of Health Sciences, University of Southern Denmark, Odense, Denmark; Department of Neurosurgery, Copenhagen University Hospital, Rigshospitalet, Copenhagen, Denmark; Department of Neurosurgery, Odense University Hospital, Odense, Denmark; Clinical Institute and BRIDGE (Brain Research-Interdisciplinary Guided Excellence), University of Southern Denmark, Odense, Denmark; Department of Oncology, Centre for Cancer and Organ Diseases, Copenhagen University Hospital, Copenhagen, Denmark; Department of Public Health, Aarhus University, Aarhus C, Denmark

**Keywords:** decisional needs, family support, high-grade glioma, patient involvement, shared decision making

## Abstract

**Background:**

High-grade gliomas are aggressive and life-threatening brain tumors. At the time of recurrence, the patients and their families need to decide on future treatment. None of the treatment options are curative, and tradeoffs between benefits and harms must be made. This study aimed to explore the patients’ and family members’ decisional needs when making the decision.

**Methods:**

We performed semi-structured individual interviews with patients and family members to explore their experiences during the decision making. A phenomenological hermeneutical analysis was conducted.

**Results:**

A total of 15 patients and 14 family members aged 22-79 years participated in the study. Most of the family members were partners to the patient. The findings were centered around three interrelated and concurrently occurring themes: (I) A patient- and family-centered decision making, including the subtheme of being a supportive family member; (II) Balanced information and a trustful professional encounter; and (III) The value of hope. We found that both the patients and family members preferred to be involved in the decision making and that a trustful relationship with the surgeon, balanced and tailored information, and sufficient time to make the decision were essential. The experience of hope had a significant influence on patients’ decisions.

**Conclusion:**

This study found that patient and family involvement, balanced information, and hope were the primary decisional needs of patients and family members at the time of recurrent high-grade glioma. Patients and family members can have different decisional needs, making individual needs assessment essential to decisional support.

High-grade gliomas (HGG) are aggressive and infiltrative primary brain tumors classified by WHO as gliomas grade III and IV.^1^ The initial treatment consists of maximum safe tumor resection followed by radiation and chemotherapy.^2–5^ However, the treatment of tumor recurrence is less evidence-based, and factors, such as tumor characteristics, tumor location, previous treatment response, and the patients’ performance state affect treatment options and outcomes.^4,6–8^ Combined with the risk of surgical complications, the treatment decision at the time of recurrence entails several uncertainties, making it a preference-sensitive decision.^8–12^

A preference-sensitive decision is characterized as a decision that must be made between two or more nearly equivalent treatment options and where patients need to consider the tradeoffs between benefits and harms.^13^ Previous research states that patients facing preference-sensitive decisions benefit from shared decision making (SDM),^13^ defined as decision making between the clinician and the patient that is based on the best available evidence and is congruent with the patient’s values and preferences.^10,14^ The SDM process can be supported by patient decision aids (PtDA).^10,11^ A PtDA can be a video-based, a paper-based, or an online decision support tool that is used as an adjunct to the clinician’s information and support during the SDM process.^11,15,16^

Recent research suggests that patients with HGG prefer an SDM approach and that decision involvement may positively affect the patients’ emotional well-being.^12,17^ In other patient populations, SDM and PtDAs have been found to improve patients’ experiences of being well informed, to decrease decisional conflict, and to support patients in expressing their needs, values, and preferences relevant to decision making.^11,15^ However, patients with HGG experience a high symptom burden, including varying degrees of cognitive impairment and reduced decision-making capacity.^18–20^ Due to these deficits, patients with HGG often rely on their families to actively participate in decision making.^21–24^

Patients who have recently been diagnosed with an HGG recurrence and their families are in a stressful situation owing to the life-threatening nature of the disease.^18,19^ Repeated surgery and palliative chemotherapy can prolong life and improve quality of life, yet the same treatments all have side effects that can decrease quality of life.^4,6–8^ Since no cure is possible, the potential benefits and harms and the values and preferences of patients and their families need to be explored before decisions are made. Therefore, it is essential to engage patients and families in SDM with sensitivity toward their preferred level of involvement and decisional needs.^25–27^ Decisional needs are defined in this study as significant factors affecting either the decision-making experiences of the patient and family or the decision itself. Decisional needs require decision support.^27^ The decisional needs of patients with HGG have not previously been explored, and the evidence on SDM for this patient population is limited.^17,27,28^ Medical specialists recognize the importance of SDM in patients with HGG, but education and recommendations concerning best clinical practice are lacking.^29^

The objective of this study was to explore the decisional needs of patients with recurrent HGG and their families to guide clinicians in providing future decision support and SDM.

## Materials and Methods

### Design

This qualitative study explored the lived experiences of HGG patients and family members without the constraint of any predefined SDM frameworks. Semi-structured interviews were carried out and analyzed using an inductive three-step Ricoeur-inspired method of analysis.^30,31^

### Participants

Eligible patients were adults diagnosed with an MRI-confirmed HGG tumor recurrence who were assessed by a multidisciplinary team (MDT) of neuro-surgeons, neuro-oncologists, neurologists, radiologists, and a clinical nurse specialist and deemed to be candidates for both surgical and oncological treatment.

The MDT assessment also functioned as a screening of the patient’s physical and cognitive state. Patients with cognitive or communicative challenges were eligible for the study if they were able to provide consent to participation and speak in complete sentences. An experienced neuro-surgeon or neuro-psychologist was consulted if the patient’s capacity to provide consent was unclear. Adult family members could participate if they were invited by the patient to take part in the decision making. Family members could be included without a participating patient. We excluded individuals who did not speak or understand Danish. The sample size was guided by considerations regarding information power in qualitative studies, such as the narrow study objective, the specificity of the sample, the quality of the interviews, and our approach to analysis.^32,33^ Based on these reflections, we assessed that a sample size between 20 and 30 participants would be appropriate to elucidate the study aim.

### Recruitment and Setting

After assessment at the MDT conference, patients were referred to a consultation with a neuro-surgeon regarding information and decision making between the following options: (1) surgical resection planned to be followed by oncological treatment; (2) oncological treatment without surgery, or (3) no active tumor treatment. Experimental treatment could be an additional fourth option in specific cases. Prior to the consultation, all patients were pre-informed about their recurrence by either an oncologist, neurologist, or neuro-surgeon. Directly following the neurosurgical consultation, patients attended a consultation with a clinical nurse specialist.

Eligible participants were informed and included by a clinical nurse specialist (H.S.E., R.G.) before the neurosurgical consultation, where the treatment options were to be discussed. Participants were consecutively recruited from Odense University Hospital between April 1, 2019 and March 31, 2020 and from Copenhagen University Hospital between December 1, 2019 and January 31, 2020.

### Data Collection

To explore the decisional needs, we developed a semi-structured interview guide (see Supplementary data) based on scientific evidence concerning SDM and PtDAs^15,17,26^ and knowledge about the natural timeline of the decision-making process at the time of recurrent HGG. The interviews were based on a few broad questions beginning with, “Can you please tell me about the day you were first informed about the recurrence?”

The interviews were carried out with an emphasis on active listening and allowing for long periods of silence and stuttering to encourage the narratives of patients with mild cognitive impairment and speech difficulties. Individual telephone interviews were conducted by H.S.E. 1-4 weeks after the treatment decision-making consultation. One interview was performed face-to-face at the hospital at the patient’s request. The interviews were audio-recorded and transcribed verbatim. Data on participant characteristics were collected and managed using REDCap electronic data capture tools hosted at OPEN (Open Patient data Explorative Network, Odense University Hospital, Region of Southern Denmark).

### Analysis

The transcripts were transferred to NVivo (QSR International Pty Ltd, version 11.4, United Kingdom) to obtain transparency and rigidity in the analytical work. The credibility of the findings was supported through investigator triangulation.^34^ Transcripts were read by three researchers independently (H.S.E., R.G., K.P.), and the coding was performed by the main author (H.S.E.) with an audit from K.P. and R.G. The subsequent interpretation and division into themes was carried out by H.S.E. and K.P. and discussed with the team. We included discussions of pre-conceptions to decrease the risk of subjective interest affecting the interpretation process.

Interviews with patients and family members were analyzed separately, enabling data triangulation, and the interpretation followed a well-defined Ricoeur-inspired three-level analysis method.^30,31^ The first level was a naïve reading of the transcripts as one whole text to attain an immediate understanding of what the text spoke about. At the second level, we carried out a structural analysis to validate or invalidate the initial impressions and gain a deeper understanding of the meaning of the text. By coding meaningful sentences and paragraphs as we reread the transcripts, we divided the text into meaning units. Meaning units that were relevant to the research question were then further condensed into themes and sub-themes. After condensing the themes, we merged the results from the analyses of patient and family interviews, respectively, into overall themes. The third level of interpretation was gaining a comprehensive understanding through critical reflection and discussion of the themes and sub-themes against relevant theory and research in the discussion section. In writing up the results, the relevant patient (P_participant number_) and family (F_participant number_) quotes were translated into English and used to support the dependability of the themes.

### Ethics

The study was conducted in accordance with the Helsinki Declaration and registered by The Danish Data Protection Agency (19/177) and the Danish National Committee on Health Research Ethics (20182000-126). Written informed consent was obtained from all participants.

## Results

### Sample Characteristics

A total of 28 patients and 21 family members were screened against the inclusion criteria. Four patients were ineligible due to aphasia or the inability to speak Danish, and two were excluded because of severe postoperative complications. A family member was included in one of the three cases where the patient was ineligible for inclusion because of severe aphasia. Two included patients showed signs of mild aphasia by occasionally searching for words during the interview. Seven patients and seven family members declined participation due to emotional distress or did not respond to our contact attempts at the time of the interviews. A total of 15 patients and 14 family members participated in the study (Table 1), with a median age of 56 years for patients and 54.5 years for family members. The family members were mainly partners. Twenty-two participants were included from Odense University Hospital and seven from Copenhagen University Hospital.

**Table 1.** Participant Characteristics

Participants (n = 29)	Patients (n = 15)	Family Members (n = 14)
Male	9	4
Female	6	10
Age (years)		
<40	–	2
40-59	10	6
60-79	5	6
Median age (range)	56.0 (40-72)	54.5 (22-79)
Diagnosis		
Glioblastoma grade IV	10	
Anaplastic astrocytoma grade III	2	
Anaplastic oligodendroglioma grade III	3	
Karnofsky score		
60-70	1	
80-100	14	
Previous recurrence	5	
Previous treatment experiences		
Resection	12	
Oncological treatment	15	
Treatment decision in this study		
Resection	11	
Oncological treatment without surgery	4	
Foregoing of active tumor treatment	0	
Family members’ relation to the patient		
Partner		11
Child or sibling		3
Highest education past primary school		
<4 years	8	6
4-7 years	5	7
≥7 years	2	1
Family status		
Living alone	4	1
Living with a partner	11	13
Having children		
None	2	2
0-17 years	4	4
18+ years	9	8

Of the 15 patients, 11 decided on resection, and 4 opted for oncological treatment without surgery. No patients decided to forego active tumor treatment, and no patients were eligible for experimental treatment or repeated radiation. Thirteen patients made their treatment decision during the consultation.

### Naïve Reading

The naïve reading provided an initial understanding of how patients and family members experienced the decision making following a diagnosis of recurrent HGG.

Patients: Learning about the recurrence was experienced as shocking news, and their first thought was: “Please, let there be a treatment option.” The patients’ fear of surgical complications was substantial, but they trusted that the surgeon would only suggest the best available treatment. Turning down a treatment option was perceived as equivalent to giving up on life. Patients emphasized that it was their body and, therefore, their decision to make. Nevertheless, it was imperative to them that their family supported the decision.

Family members: The recurrence evoked thoughts about death and worries about the future. They underscored that a treatment decision should always be the patient’s decision even though it affected the whole family, and they felt responsible for supporting the patient all the way. They expressed a high level of trust in the surgeon’s recommendations and valued surgeons treating the patient with kindness and respect. They were aware of their vital role in the patient’s life and decision making, and they wanted the healthcare professionals to acknowledge their significance.

### Structural Analysis

**Figure 1.**
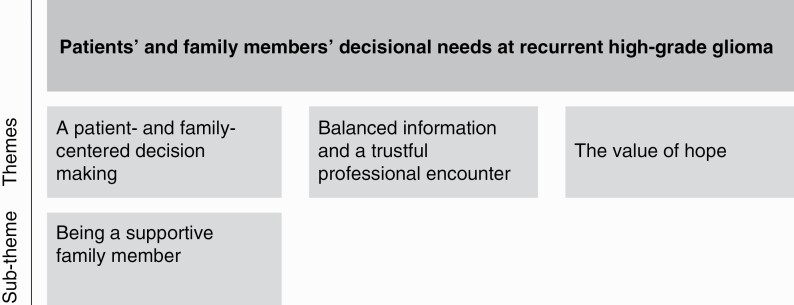
Patients’ and family members’ decisional needs at recurrent high-grade glioma.

Through the structural analysis, we identified three interrelated and concurrently occurring themes shared by both patients and families as well as one subtheme specific to family members: (I) A patient- and family-centered decision making, including the subtheme of being a supportive family member, (II) Balanced information and a trustful professional encounter; and (III) The value of hope (Figure 1).

#### Theme I: A patient- and family-centered decision making—

The patient’s preference to be involved in the decision making regarding their treatment and care was evident and persistent.

It’s about YOUR life, and of course YOU should make the decision. The doctor can’t do it for you. Well, they can guide you and give you some advice; tell you whether they think it’s a good idea. But in the end, it’s only me. (P21)

Despite preferring involvement, some patients experienced the decision-making responsibility as a burden, and they felt uncertain about their decision even after the treatment had been carried out.

It would have been easier for me [if the doctor made the decision] because I’m always wondering, did I do the right thing? Or what could I have done? But I’ll never know. (P18)

Patients and family members expressed that the family’s participation in the decision making was imperative and that family played a significant role both practically and emotionally. This was especially evident for those patients who experienced aphasia or other cognitive impairments and were therefore dependent on their family to speak for them or to assist them in different ways to make a decision.

We talked about it. Or I talked about it, and he [patient with aphasia] nodded or shook his head and said, “There’s no doubt.” (F05)

Some patients made their decision almost instantly, but they still highlighted their family’s implicit support.

It meant a lot to me knowing that I made the decision and she [patient’s wife] was supportive of it and understood. (P17)

Both the patients and family members agreed that even though the decision could have a tremendous impact on the family’s life, it was the patient who had the final word in making the decision.

In the very end, it’s his decision. We can’t say, “Do this,” if he doesn’t want to. (F16)

*Subtheme: Being a supportive family member:* Watching their loved ones suffer caused the family members to put aside their feelings of grief and worry and, instead, focus on supporting the patient and keeping the family together. The family member felt obliged to keep track of everything and comfort the patient in the decision-making situation.

And if there were things during the consultation that she [the patient] didn’t say, then I would assist, enhancing discussions that I found relevant but which she, for some reason, didn’t mention on her own. Or I’d listen and hold her hand when she needed it. (F14)

The family members were aware of their vital role in the patients’ decision making. They emphasized the importance of being recognized by the health professionals as equal members of the decision-making team.

I actually had the feeling that the three of us, including the surgeon, were pretty much on the same team and together we should find the best possible solution to this. (F19)

Some family members, on the other hand, expressed their frustrations of not feeling acknowledged.

Well, I feel that in many ways the family doesn’t count in the health care professionals’ universe. (F17)

#### Theme II: Balanced information and a trustful professional encounter—

Having a trustful relationship with the surgeon could directly influence the participants’ decision making by causing them to be more or less likely to choose surgery. A previous positive collaboration with the surgeon added cohesion and comfort to the current distressing situation.

If I hadn’t had good chemistry with the doctor or hadn’t felt safe, then I don’t know. Then I am not sure I would have chosen the operation. (P18)

Participants expressed a need to rely on the surgeon’s expertise and recommendations to manage the uncertainty related to the decision making. Some chose to delegate the decision-making responsibility to the surgeon by following the recommendations without further considerations.

If they recommend that I get surgery, that’s what I will choose. (P16)

The way the surgeons balanced their information and the words used to describe the tumor or surgical procedure affected participants’ risk assessment and treatment decision.

Well, they said the procedure was quite minor and easy. That the tumor was just a little round ball without arms. (P12)

The participants stressed that receiving adequate information delivered in an understandable way was vital to making a decision. The preferences regarding the type and amount of information differed, highlighting the importance of individually tailored information. For some participants, visual information such as watching MRI scans provided a more comprehensive understanding of the disease progression and the (non-)response to treatment.

I have asked to see the MRI scans every time. I wanted to see the tumor with my own eyes. (P17)

On the other hand, statistical information was often experienced as difficult to relate to and therefore needed to be followed up with individually oriented information.

We could have used some more personal guidance as well, instead of just all the hard facts [statistics]. (F13)

To assimilate a lot of information within a short time was challenging for both patients and family members, especially in cases where the patient had cognitive impairments.

Well, first he got the message [about progression] and right afterward, a lot of information. At that point, I could have used a little break. And THEN they could have provided us with the information we needed to make the decision. (F13)

On the other hand, having sufficient time to ask questions and to further discuss the treatment options was highlighted as valuable.

It seemed like she [the surgeon] took the time to let my mother ask all of her questions. We didn’t feel anything was missing or that there were things we couldn’t say. We talked about everything, which was nice because it was a stressful situation overall. (F14)

In cases where the patient and family did not understand the surgeon’s information, the clinical nurse specialist played an essential role in repeating and explaining it.

They [doctors in general] are all right, but it seems like these doctors know a lot about the treatment. And then afterward, you talk to the nurse and get a chance to find out what the doctor was actually saying. (P01)

#### Theme III: The value of hope—

The feeling of hope was a significant factor in the decision making. Regardless of how hopeless the situation seemed, the participants emphasized that the clinicians should never take away a patient’s hope.

[Some clinicians] take away the sparkle and hope from a person—even before they engage in a decent conversation, and I just feel that you should NEVER do that. You have to say that this is what it [the prognosis] looks like and this is what we can do for you. (F15)

Hope was often linked to the availability of further active treatment, indicating that the option of foregoing treatment was considered to result from a lack of other options rather than being an option in itself. Surgery, in particular, was perceived as being equivalent to hope.

I mean, of course, it’s not good that it’s growing, but the fact that there’s still something that can be done [surgery]—that’s what is holding me up; that it is still possible to do something, so hopefully I can live a little longer. (P21)

As a result, the decision to decline surgery due to the fear of risks or previous experiences of complications caused the participants great distress. Some participants even expressed that saying no to surgery was the same as saying no to life.

If you say, “no thank you” [to the operation], well then you really don’t have anything left. (F10)

When asked directly, the participants valued quality of life over prolonging life, but this was not necessarily reflected in their treatment decisions. Instead, the hope of prolonging life affected patients’ and family members’ willingness to accept or reject the potential risks related to the treatment. Most participants articulated that they were aware of the risks, and some expressed that the knowledge of possible complications was a heavy burden to them. Nevertheless, they felt they had no choice.

I was so afraid of ending up like a vegetable, not being able to do anything. And the doctor couldn’t make any promises, but at the same time, I knew that if I didn’t accept the treatment, I might end up like this anyway. I had to choose the treatment. (P18)

Others found that the hope of maintaining existing cognitive or physical functions was more critical than the diminishing hope of prolonging life.

His cognitive function is damaged pretty badly, I would say, but he still has his physical abilities and he loves to work in his garden. If those abilities were to get hampered by the operation … we just couldn’t take the risk. (F03)

In some cases, the values surrounding the options differed between patients and family members. This could occur in situations where the patient valued the hope of life prolongation over the risk of complications, whereas the family member valued the patient’s well-being above all. These situations caused distress for all those involved and required specific attention from the professional team.

I would probably say stop before he would. But it’s the thing about watching someone suffer or being in the situation yourself and still having hope. (F13)

## Discussion

To the best of our knowledge, this is the first study to identify the decisional needs of patients with HGG and their family members at the time of recurrent HGG. We found that a trustful relationship with the surgeon, the surgeon’s balancing of information, and the experience of hope affected the patients’ decision making. Both patients and family members preferred being involved, and the family played an essential, supportive role. This leads to the following points of discussion.

Patients and family members emphasized that patient involvement in decision making is imperative and that the patient should be at the center of the decision-making team. These findings are supported by the results of a recent systematic review, which concludes that patients with HGG prefer to be involved in decision making.^17^ To be actively involved in decision making requires that patients have a certain level of decision-making capacity. This implies the ability to understand the information provided, appreciate the situation and the consequences of the different options, weigh the benefits and harms related to the options, and communicate their preferred decision. Unfortunately, in patients with HGG, cognitive impairments, speech difficulties, and decreased decision-making capacity are frequent.^21,22^ This challenges patient involvement and SDM for this patient group. Research of other patient populations suffering from cognitive impairments suggests that using written information, pictures, and simple language as well as providing time to reflect on the decision and encouraging family participation are ways to support the involvement of patients with cognitive or communicative challenges in decision making.^35–37^ These suggestions reflect our findings, indicating that the involvement of patients with HGG and cognitive impairments may be supported in several ways. One way could be the use of PtDAs, which incorporate many of the above-mentioned factors and have been found to improve patient involvement and support patient-clinician communication in other patient populations.^11,15^ The involvement of family is another critical factor in supporting patients with HGG in decision making. The family members experience a high level of responsibility toward the patient in both decision making and daily life.^18,38–40^ Family members are aware of their significant role, and they desire recognition and support from the health professionals.

Participants expressed that hope was essential and that the clinicians should communicate prognostic information in a way that preserved hope. This correlates with previous findings of hope being an important factor in the lives of patients and families living with an HGG diagnosis.^18,19,41^ Most patients in this study expressed that they valued quality of life more than prolonging life. Nevertheless, no patients decided to forego life-prolonging treatment, even in cases where the treatment was associated with a potential decrease in their quality of life.

The decisional needs of patients and family members at the time of recurrent HGG mirrored the description of decisional needs in the Ottawa Decision Support Framework (ODSF).^27,42^ The ODSF proposes eight areas of decisional needs: the type and timing of the decision, the patient’s decisional stage, decisional conflict and uncertainty, inadequate knowledge, unrealistic expectations, unclear values, inadequate support, and the patient’s personal and clinical characteristics.^27,42^ Concerning the type and timing of the decision, HGG patients and their families were in a stressful situation that was characterized by powerful emotions. They experienced decisional conflict concerning unclear values toward life prolongation vs quality of life. We also identified conflicting values and preferences when the family member preferred ending treatment before the patient did. Previous research suggests that these different values and preferences could be founded in variances related to prognostic awareness^43–45^ and highlights the importance of clarifying values and expectations for both patients and their families. Adequate knowledge about treatment options and the patient’s situation was a decisional need for all participants, and the surgeon’s communication skills and balancing of information were crucial. As the above discussion implies, the ODSF may be a valuable conceptual model to support SDM in patients with HGG by identifying the decisional needs of patients and their families.

Some participants felt overwhelmed by the intensity of receiving a lot of information and then having to make a treatment decision during just one consultation. In addition, patients and family members described the clinical nurse specialist as being essential to the process of fully comprehending the surgeon’s information. This opens up considerations about an interprofessional SDM approach.^12,46^ Interprofessional SDM is characterized by the participation of at least two health care professionals from different professions and allows the decision making to proceed over more than one consultation, for example, a consultation with a nurse providing decision coaching in relation to the consultation with the surgeon.^46,47^ This may help to better prepare the HGG patients and family members to discuss the decision, counteract information overload during the consultation, and provide improved decision support.^47,48^

### Methodological Considerations

The transferability of this study is limited to patients with HGG with a performance status compatible with neurosurgical and oncological treatment, and patients who want to continue active treatment. Additionally, since the study was carried out within the Danish healthcare system, transferability to other cultural settings may be limited. We excluded patients with severe aphasia, and the decision-making experiences of this sub-population are therefore only presented through interviews with family. Fourteen eligible participants did not respond to our contact attempts or declined participation. The primary reason for declining was psychological distress, indicating that the study failed to include the most fragile patients and family members.

A strength of the study is the inclusion of patients with cognitive and communicative impairments, which increases transferability to the actual patient population. Tailored interview and communication techniques in combination with data triangulation were used to increase credibility. In addition, investigator triangulation, focus on researcher pre-conceptions, and the transparent stepwise analysis strengthen the confirmability and dependability of the results.

## Conclusions

This study found that even though most patients and family members preferred to be involved in the decision making following recurrent HGG, some patients preferred to leave the decision to the surgeon. This points out the importance of assessing individual needs when offering decision support. Experiencing a trustful relationship with the surgeon, receiving balanced and tailored information, and having sufficient time to discuss the treatment options were essential decisional needs shared by both patients and family members. Additionally, hope had a significant impact on decision making.

Though many of the identified needs were similar for patients and family members, the family also had unique needs concerning being acknowledged by the health professionals. Patients and family members did not necessarily share the same values regarding the treatment options, making value clarification a significant decisional need for both patients and family members.

Further research focusing on involvement of other interprofessional team members, such as the clinical nurse specialist, or the development of PtDAs is essential. Additionally, knowledge about how previous treatment experiences, prognostic awareness, and palliative care options may influence HGG patients’ decision making is needed to understand decisional needs and improve decisional support to patients with recurrent HGG and their families.

## Supplementary Material

npac046_suppl_Supplementary_Data
