# Supplementary material for: Decisional needs of patients with recurrent high-grade glioma and their families
Source: Neurooncol Pract. 2022 Jun 11;9(5):402–10. doi: 10.1093/nop/npac046 (PMC9476966; doi:10.1093/nop/npac046)
Supplement: npac046_suppl_Supplementary_Data [file npac046_suppl_supplementary_data.docx]

| Research question: | What are the involvement preferences and decisional needs of patients with recurrent HGG and their family members and which factors affect the decision? |
| --- | --- |
| Overall questions | **Follow-up questions** |
| Can you please tell me about the day you were first informed about the recurrence? | - How were you informed? Where? By whom?  - What were you told?  - What did you think/feel/do?  - Were you prepared that the tumor would someday start growing again? |
| Can you please tell me about the days starting from when you were informed about the recurrence until the consultation with the surgeon where you discussed the treatment options? | - Which considerations or worries did you have regarding the consultation?  - What did you think/feel/do?  - How did you prepare for the consultation?  - Where did you find support?  - Was there any information or support that you needed but did not receive?  - Did you have any treatment preferences? |
| Can you please tell me about the consultation and how the treatment decision was made? | - Who participated in the consultation?  - What options were you informed about?  - Was it clear to you that you had more than one option?  - Had you made your decision before the consultation?  - Did the consultation change your decision?  - What was particularly helpful to you when making the decision?  - Would you have made the same decision today? Why/why not? |
| When thinking about the process as a whole, how do you think it could have been improved? | - Information/support/anything else? |

Supplementary data: The interview guide
